# Supplementary material for: Multidimensional correlates of psychological stress: Insights from traditional statistical approaches and machine learning using a nationally representative Canadian sample
Source: PLoS One. 2025 May 13;20(5):e0323197. doi: 10.1371/journal.pone.0323197 (PMC12074393; doi:10.1371/journal.pone.0323197)
Supplement: S1 Table — A list of variables from the CCHS that were used in this analysis, including the name of variable, original wording (for items asked in the CCHS-MH) or concept (for derived variables), and the scoring used for the present study. (DOCX) [file pone.0323197.s001.docx]

**Table S1 –****Detailed Variable Description.** A list of variables from the CCHS that were used in this analysis, including the name of variable, original wording (for items asked in the CCHS-MH) or concept (for derived variables), and the scoring used for the present study.

| **Category** | **Variable Name  (CCHS-MH Variable Label)** | **Question Text / Concept** | **Original Coding** | **Current Study Coding** |
| --- | --- | --- | --- | --- |
| Demographic Factors | Age  (DHHGAGE) | Age - grouped variable | 1 ~ 15 TO 19 YEARS  2 ~ 20 TO 24 YEARS  3 ~ 25 TO 29 YEARS  4 ~ 30 TO 34 YEARS  5 ~ 35 TO 39 YEARS  6 ~ 40 TO 44 YEARS  7 ~ 45 TO 49 YEARS  8 ~ 50 TO 54 YEARS  9 ~ 55 TO 59 YEARS  10 ~ 60 TO 64 YEARS  11 ~ 65 TO 69 YEARS  12 ~ 70 TO 74 YEARS  13 ~ 75 TO 79 YEARS  14 ~ 80 YEARS OR OLDER | 13 ~ 20 TO 24 YEARS  12 ~ 25 TO 29 YEARS  11 ~ 30 TO 34 YEARS  10 ~ 35 TO 39 YEARS  9 ~ 40 TO 44 YEARS  8 ~ 45 TO 49 YEARS  7 ~ 50 TO 54 YEARS  6 ~ 55 TO 59 YEARS  5 ~ 60 TO 64 YEARS  4 ~ 65 TO 69 YEARS  3 ~ 70 TO 74 YEARS  2 ~ 75 TO 79 YEARS  1 ~ 80 YEARS OR OLDER |
| Demographic Factors | Body Mass Index  (HWTGBMI) | Body Mass Index (BMI) / self-report | 14.00 - 54.96 | 14.00 - 54.96 |
| Demographic Factors | Dwelling type  (DHHGDWE) | Dwelling Type | 1 ~ SINGLE DETACHED 2 ~ APARTMENT  3 ~ OTHER | “SINGLE DETACHED” “APARTMENT”  “OTHER” |
| Demographic Factors | Education  (EDUDR04) | Highest level of education | 1 ~ LESS THAN SECONDARY SCHOOL GRADUATION  2 ~ SECONDARY SCHOOL GRADUATION  3 ~ SOME POST-SECONDARY  4 ~ POST-SECONDARY GRADUATION | “LESS THAN SECONDARY SCHOOL GRADUATION”<  “SECONDARY SCHOOL GRADUATION” <  “SOME POST-SECONDARY” < “POST-SECONDARY GRADUATION” |
| Demographic Factors | Employment  (GEN_08) | Have you worked at a job or business at any time in the past 12 months? | 1 ~ YES  2 ~ NO | 1 ~ YES  0 ~ NO |
| Demographic Factors | Household size  (DHHGHSZ) | Household Size | 1 ~ 1 PERSON  2 ~ 2 PERSONS  3 ~ 3 PERSONS  4 ~ 4 PERSONS  5 ~ 5 OR MORE PERSONS | 1 ~ 1 PERSON  2 ~ 2 PERSONS  3 ~ 3 PERSONS  4 ~ 4 PERSONS  5 ~ 5 OR MORE PERSONS |
| Demographic Factors | Household type  (DHHGLVG) | Living/Family Arrangement of Selected Respondent | 1 ~ UNATTACHED INDIVIDUAL LIVING ALONE  2 ~ UNATTACHED INDIVIDUAL LIVING WITH OTHERS  3 ~ LIVING WITH SPOUSE / PARTNER  4 ~ PARENT LIVING W/SPOUSE/PARTN., CHILDREN  5 ~ SINGLE PARENT LIVING WITH CHILDREN  6 ~ CHILD LIVING W/ONE PARENT W/WO SIBLINGS  7 ~ CHILD LIVING W/TWO PARENTS W/WO SIBLINGS  8 ~ OTHER | “UNATTACHED INDIVIDUAL LIVING ALONE”  “UNATTACHED INDIVIDUAL LIVING WITH OTHERS”  “LIVING WITH SPOUSE / PARTNER”  “PARENT LIVING W/SPOUSE/PARTN., CHILDREN”  “SINGLE PARENT LIVING WITH CHILDREN”  “CHILD LIVING W/ONE PARENT W/WO SIBLINGS”  “CHILD LIVING W/TWO PARENTS W/WO SIBLINGS”  “OTHER” |
| Demographic Factors | Immigrant status  (SDCFIMM) | Immigrant | 1 ~ YES  2 ~ NO | 1 ~ YES  0 ~ NO |
| Demographic Factors | Income  (INCDRPR) | Household income distribution - provincial level | 1 ~ DECILE 1  2 ~ DECILE 2  3 ~ DECILE 3  4 ~ DECILE 4  5 ~ DECILE 5  6 ~ DECILE 6  7 ~ DECILE 7  8 ~ DECILE 8  9 ~ DECILE 9  10 ~ DECILE 10 | 10 ~ DECILE 1  9 ~ DECILE 2  8 ~ DECILE 3  7 ~ DECILE 4  6 ~ DECILE 5  5 ~ DECILE 6  4 ~ DECILE 7  3 ~ DECILE 8  2 ~ DECILE 9  1 ~ DECILE 10 |
| Demographic Factors | Marital status  (DHHGMS) | Marital Status | 1 ~ MARRIED  2 ~ COMMON-LAW  3 ~ WIDOWED  4 ~ DIVORCED/SEPARATED  5 ~ SINGLE | “MARRIED”  “COMMON-LAW”  “WIDOWED”  “DIVORCED/SEPARATED”  “SINGLE” |
| Demographic Factors | Province of residence  (GEO_PRV) | Province of residence of respondent | 10 ~ NEWFOUNDLAND AND LABRADOR  11 ~ PRINCE EDWARD ISLAND  12 ~ NOVA SCOTIA  13 ~ NEW BRUNSWICK  24 ~ QUEBEC  35 ~ ONTARIO  46 ~ MANITOBA  47 ~ SASKATCHEWAN  48 ~ ALBERTA  59 ~ BRITISH COLUMBIA | "NEWFOUNDLAND AND LABRADOR"  "PRINCE EDWARD ISLAND"  "NOVA SCOTIA"  "NEW BRUNSWICK"  "QUEBEC"  "ONTARIO"  "MANITOBA"  "SASKATCHEWAN"  "ALBERTA"  "BRITISH COLUMBIA" |
| Demographic Factors | Sex  (DHH_SEX) | Interviewer: Enter the respondent's sex. If necessary, ask: Is respondent male or female? | 1 ~ MALE  2 ~ FEMALE | 0 ~ MALE  1 ~ FEMALE |
| Demographic Factors | Student status  (SDC_8) | Are you currently attending a school, college, cegep or university? | 1 ~ YES  2 ~ NO | 1 ~ YES  0 ~ NO |
| Demographic Factors | Visible minority status  (SDCGCGT) | Culture / Race Flag | 1 ~ WHITE  2 ~ NON-WHITE (ABORIGIN. OR VIS. MIN.) | 0 ~ WHITE  1 ~ NON-WHITE (ABORIGIN. OR VIS. MIN.) |
| Health Behaviours | Alcohol abuse or dependence (AUDDY) | Alcohol dependence - Algorithm | 1 ~ YES  2 ~ NO | 1 ~ YES  0 ~ NO |
| Health Behaviours | Drug abuse or dependence (SUDDY) | Drug abuse or dependence (including cann) - 12 m | 1 ~ YES  2 ~ NO | 1 ~ YES  0 ~ NO |
| Health Behaviours | Frequency of drinking (AUD_02) | During the past 12 months, how often did you drink alcoholic  beverages? | 1 ~ LESS THAN ONCE A MONTH  2 ~ ONCE A MONTH  3 ~ 2 TO 3 TIMES A MONTH  4 ~ ONCE A WEEK  5 ~ 2 TO 3 TIMES A WEEK  6 ~ 4 TO 6 TIMES A WEEK  7 ~ EVERY DAY | 1 ~ LESS THAN ONCE A MONTH  2 ~ ONCE A MONTH  3 ~ 2 TO 3 TIMES A MONTH  4 ~ ONCE A WEEK  5 ~ 2 TO 3 TIMES A WEEK  6 ~ 4 TO 6 TIMES A WEEK  7 ~ EVERY DAY |
| Health Behaviours | Illicit drug use (SUDDYID) | Any drug use (excl cann use) - 12 m | 1 ~ YES  2 ~ NO | 1 ~ YES  0 ~ NO |
| Health Behaviours | Level of insomnia  (GEN_04) | How often do you have trouble going to sleep or staying asleep? | 1 ~ NONE OF THE TIME  2 ~ A LITTLE OF THE TIME  3 ~ SOME OF THE TIME  4 ~ MOST OF THE TIME  5 ~ ALL OF THE TIME | 1 ~ NONE OF THE TIME  2 ~ A LITTLE OF THE TIME  3 ~ SOME OF THE TIME  4 ~ MOST OF THE TIME  5 ~ ALL OF THE TIME |
| Health Behaviours | Smoking status  (SMKDSTY) | Type of smoker | 1 ~ DAILY SMOKER  2 ~ OCCASIONAL SMOKER (FORMER DAILY SMOKER)  3 ~ ALWAYS AN OCCASIONAL SMOKER  4 ~ FORMER DAILY SMOKER  5 ~ FORMER OCCASIONAL SMOKER  6 ~ NEVER SMOKED | "DAILY SMOKER"  "OCCASIONAL SMOKER (FORMER DAILY SMOKER)"  "ALWAYS AN OCCASIONAL SMOKER"  "FORMER DAILY SMOKER"  "FORMER OCCASIONAL SMOKER"  "NEVER SMOKED" |
| Health Behaviours | Weekly hours of moderate or vigorous physical activity (PHSGAPA) | Moderate/vigorous physical activity -average/hours - 7 D | 1. - 13.50 ~ HOURS   14 ~ 14 HOURS OF MORE | 5 ~ 0 Hours  4 ~ 0.1 – 2.49 Hours  3 ~ 2.50 – 4.99 Hours  2 ~ 5 - 7.49 Hours  1 ~ 7.50 – 9.99 Hours  0 ~ 10+ Hours |
| Life Adversity | Early life adversity - Sum Score  (CEXDNUM) | Number of types of childhood maltreatment experienced | 0-6 NUMBER OF TYPES | 0-6 |
| Life Adversity | Recent life events - Family problems  (CWP_07) | During the past 12 months, did you come into contact with the police for reasons related to a family member's problems with their emotions, mental health or use of alcohol or drugs? | 1 ~ YES  2 ~ NO | 1 ~ YES  0 ~ NO |
| Life Adversity | Recent life events - Unmet needs  (PNCDNEED) | Overall Perceived Need in Past 12 Months | 1 ~ NO PERCEIVED NEED  2 ~ ALL PERCEIVED NEEDS MET  3 ~ PERCEIVED NEEDS PARTIALLY MET  4 ~ PERCEIVED NEEDS NOT MET | 0 ~ NO PERCEIVED NEED & ALL PERCEIVED NEEDS MET  1~ PERCEIVED NEEDS PARTIALLY MET & PERCEIVED NEEDS NOT MET |
| Life Adversity | Recent life events - Victim of a crime  (CWP_03) | During the past 12 months, did you come into contact with the police: as a victim of a crime? | 1 ~ YES  2 ~ NO | 1 ~ YES  0 ~ NO |
| Life Adversity | Recent life Events - Witness a crime  (CWP_04) | During the past 12 months, did you come into contact with the police: as a witness to a crime? | 1 ~ YES  2 ~ NO | 1 ~ YES  0 ~ NO |
| Mental Health | Anxiety disorder  (CCC_290) | Do you have an anxiety disorder such as a phobia, obsessive-compulsive disorder or a panic disorder? | 1 ~ YES  2 ~ NO | 1 ~ YES  0 ~ NO |
| Mental Health | Attention deficit disorder  (CCC_332) | Do you have Attention Deficit Disorder? | 1 ~ YES  2 ~ NO | 1 ~ YES  0 ~ NO |
| Mental Health | Bipolar disorder  (BIPDL) | Bipolar Disorder - Algorithm - life | 1 ~ YES  2 ~ NO | 1 ~ YES  0 ~ NO |
| Mental Health | Generalized anxiety disorder  (GADDDY) | Generalized anxiety disorder - algorithm - 12 m | 1 ~ YES  2 ~ NO | 1 ~ YES  0 ~ NO |
| Mental Health | Hypomania  (HYPDEY) | Hypomania - Algorithm - 12 m | 1 ~ YES  2 ~ NO | 1 ~ YES  0 ~ NO |
| Mental Health | Learning disability  (CCC_331) | Remember, we're interested in conditions diagnosed by a health professional and are expected to last or have already lasted 6 months or more. Do you have a learning disability? | 1 ~ YES  2 ~ NO | 1 ~ YES  0 ~ NO |
| Mental Health | Major depressive episode  (DEPDDY) | Major Depressive Episode - Algorithm - 12 m | 1 ~ YES  2 ~ NO | 1 ~ YES  0 ~ NO |
| Mental Health | Mania  (MIADEY) | Mania - 2002 Algorithm - 12 m | 1 ~ YES  2 ~ NO | 1 ~ YES  0 ~ NO |
| Mental Health | PTSD  (CCC_311) | Do you have post-traumatic stress disorder? | 1 ~ YES  2 ~ NO | 1 ~ YES  0 ~ NO |
| Mental Health | Suicidal thoughts  (DEPFSLT) | Suicide - thought - life | 1 ~ YES  2 ~ NO | 1 ~ YES  0 ~ NO |
| Physical Health | Arthritis  (CCC_051) | Remember, we’re interested in conditions diagnosed by a health professional and are expected to last or have already lasted 6 months or more. Do you have arthritis, excluding fibromyalgia? | 1 ~ YES  2 ~ NO | 1 ~ YES  0 ~ NO |
| Physical Health | Asthma  (CCC_031) | Do you have asthma? | 1 ~ YES  2 ~ NO | 1 ~ YES  0 ~ NO |
| Physical Health | Back problems  (CCC_061) | Do you have back problems, excluding fibromyalgia and arthritis? | 1 ~ YES  2 ~ NO | 1 ~ YES  0 ~ NO |
| Physical Health | Bowel disorders  (CCC_171) | Do you have a bowel disorder such as Crohn’s Disease, ulcerative colitis, Irritable Bowel Syndrome or bowel incontinence? | 1 ~ YES  2 ~ NO | 1 ~ YES  0 ~ NO |
| Physical Health | Chronic fatigue  (CCC_251) | Remember, we're interested in conditions diagnosed by a health professional and are expected to last or have already lasted 6 months or more. Do you have chronic fatigue syndrome? | 1 ~ YES  2 ~ NO | 1 ~ YES  0 ~ NO |
| Physical Health | Current cancer  (CCC_131) | Do you have cancer? | 1 ~ YES  2 ~ NO | 1 ~ YES  0 ~ NO |
| Physical Health | Diabetes  (CCC_101) | Do you have diabetes? | 1 ~ YES  2 ~ NO | 1 ~ YES  0 ~ NO |
| Physical Health | Difficulty household responsibilities  (DASG02) | In the last 30 days, how much difficulty did you have in taking care of your household responsibilities? | 1 ~ NONE  2 ~ MILD  3 ~ MODERATE  4 ~ SEVERE/EXTREME/CANNOT DO | 1 ~ NONE  2 ~ MILD  3 ~ MODERATE  4 ~ SEVERE/ EXTREME/ CANNOT DO |
| Physical Health | Difficulty standing  (DASG01) | In the last 30 days, how much difficulty did you have in standing for long periods such as 30 minutes? | 1 ~ NONE  2 ~ MILD  3 ~ MODERATE  4 ~ SEVERE/EXTREME/CANNOT DO | 1 ~ NONE  2 ~ MILD  3 ~ MODERATE  4 ~ SEVERE/ EXTREME/ CANNOT DO |
| Physical Health | Difficulty walking  (DAS_07) | In the last 30 days, how much difficulty did you have in walking a long distance such as a kilometre (or 0.6 miles)? | 1 ~ NONE  2 ~ MILD  3 ~ MODERATE  4 ~ SEVERE  5 ~ EXTREME/CANNOT DO | 1 ~ NONE  2 ~ MILD  3 ~ MODERATE  4 ~ SEVERE  5 ~ EXTREME/CANNOT DO |
| Physical Health | Heart disease  (CCC_121) | Do you have heart disease? | 1 ~ YES  2 ~ NO | 1 ~ YES  0 ~ NO |
| Physical Health | High blood pressure  (CCC_071) | Remember, we’re interested in conditions diagnosed by a health professional and are expected to last or have already lasted 6 months or more. Do you have high blood pressure? | 1 ~ YES  2 ~ NO | 1 ~ YES  0 ~ NO |
| Physical Health | Migraines  (CCC_081) | Remember, we're interested in conditions diagnosed by a health professional and are expected to last or have already lasted 6 months or more. Do you have migraine headaches? | 1 ~ YES  2 ~ NO | 1 ~ YES  0 ~ NO |
| Physical Health | Previous cancer  (CCC_132) | Have you ever been diagnosed with cancer? | 1 ~ YES  2 ~ NO | 1 ~ YES  0 ~ NO |
| Physical Health | Self-perceived health  (GEN_01) | In general, would you say your health is...? | 1 ~ EXCELLENT  2 ~ VERY GOOD  3 ~ GOOD  4 ~ FAIR  5 ~ POOR | 1 ~ EXCELLENT  2 ~ VERY GOOD  3 ~ GOOD  4 ~ FAIR  5 ~ POOR |
| Physical Health | Stroke  (CCC_151) |  | 1 ~ YES  2 ~ NO | 1 ~ YES  0 ~ NO |
| Psychological Factors | Coping on a daily basis  (STS_2) | In general, how would you rate your ability to handle the day-to-day demands in your life, for example, handling work, family and volunteer responsibilities? Would you say your ability is…? | 1 ~ EXCELLENT  2 ~ VERY GOOD  3 ~ GOOD  4 ~ FAIR  5 ~ POOR | 1 ~ EXCELLENT  2 ~ VERY GOOD  3 ~ GOOD  4 ~ FAIR  5 ~ POOR |
| Psychological Factors | Coping skill  (STS_5) | When faced with this source of stress (Author’s Note – this references Greatest Source of Stress / STS_3), you have the personal ability to deal with the situation. Do you...? | 1 ~ STRONGLY AGREE  2 ~ AGREE  3 ~ NEITHER AGREE NOR DISAGREE  4 ~ DISAGREE  5 ~ STRONGLY DISAGREE | 1 ~ STRONGLY AGREE  2 ~ AGREE  3 ~ NEITHER AGREE NOR DISAGREE  4 ~ DISAGREE  5 ~ STRONGLY DISAGREE |
| Psychological Factors | Coping with crisis  (STS_1) | In general, how would you rate your ability to handle unexpected and difficult problems, for example, a family or personal crisis? Would you say your ability is...? | 1 ~ EXCELLENT  2 ~ VERY GOOD  3 ~ GOOD  4 ~ FAIR  5 ~ POOR | 1 ~ EXCELLENT  2 ~ VERY GOOD  3 ~ GOOD  4 ~ FAIR  5 ~ POOR |
| Psychological Factors | Difficulty concentrating  (DAS_06) | In the last 30 days, how much difficulty did you have in concentrating on doing something for 10 minutes? | 1 ~ NONE  2 ~ MILD  3 ~ MODERATE  4 ~ SEVERE  5 ~ EXTREME/CANNOT DO | 1 ~ NONE  2 ~ MILD  3 ~ MODERATE  4 ~ SEVERE  5 ~ EXTREME/CANNOT DO |
| Psychological Factors | Emotional impact of health  (DAS_05) | In the last 30 days, how much have you been emotionally affected by your health problems? | 1 ~ NONE  2 ~ MILD  3 ~ MODERATE  4 ~ SEVERE  5 ~ EXTREME/CANNOT DO | 1 ~ NONE  2 ~ MILD  3 ~ MODERATE  4 ~ SEVERE  5 ~ EXTREME/CANNOT DO |
| Psychological Factors | Greatest source of stress  (STS_3) | Thinking about stress in your day-to-day life, what would you say is the most important thing contributing to feelings of stress you may have? | 1~ TIME PRESSURES / NOT ENOUGH TIME  2 ~ OWN PHYSICAL HEALTH PROBLEM OR CONDITION  3 ~ OWN EMOTIONAL OR MENTAL HEALTH PROBLEM  4 ~ FINANCIAL SITUATION  5 ~ OWN WORK SITUATION  6 ~ SCHOOL  7 ~ EMPLOYMENT STATUS  8 ~ CARING FOR - OWN CHILDREN  9 ~ CARING FOR - OTHERS  10 ~ OTHER PERSONAL OR FAMILY RESPONSIBILITY  11 ~ PERSONAL RELATIONSHIPS  12 ~ DISCRIMINATION  13 ~ PERSONAL AND FAMILY'S SAFETY  14 ~ HEALTH OF FAMILY MEMBERS  15 ~ OTHER  16 ~ NOTHING  17 ~ LOSS OF LOVED ONE | "TIME PRESSURES / NOT ENOUGH TIME"  "OWN PHYSICAL HEALTH PROBLEM OR CONDITION"  "OWN EMOTIONAL OR MENTAL HEALTH PROBLEM"  "FINANCIAL SITUATION"  "OWN WORK SITUATION"  "SCHOOL"  "EMPLOYMENT STATUS"  "CARING FOR - OWN CHILDREN"  "CARING FOR - OTHERS"  "OTHER PERSONAL OR FAMILY RESPONSIBILITY"  "PERSONAL RELATIONSHIPS"  "DISCRIMINATION"  "PERSONAL AND FAMILY'S SAFETY"  "HEALTH OF FAMILY MEMBERS"  "OTHER"  "NOTHING"  "LOSS OF LOVED ONE" |
| Psychological Factors | Life satisfaction  (GEN_02A2) | Using a scale of 0 to 10 where 0 means "Very dissatisfied" and 10 means "Very satisfied", how do you feel about your life as a whole right now? | 0 ~ VERY DISSATISFIED  1 ~ 1  2 ~ 2  3 ~ 3  4 ~ 4  5 ~ 5  6 ~ 6  7 ~ 7  8 ~ 8  9 ~ 9  10 ~ VERY SATISFIED | 0 ~ VERY SATISFIED  1 ~ 9  2 ~ 8  3 ~ 7  4 ~ 6  5 ~ 5  6 ~ 4  7 ~ 3  8 ~ 2  9 ~ 1  10 ~ VERY DISSATISFIED |
| Social Factors | Community belonging  (GEN_10) | How would you describe your sense of belonging to your local community? Would you say it is...? | 1 ~ VERY STRONG  2 ~ SOMEWHAT STRONG  3 ~ SOMEWHAT WEAK  4 ~ VERY WEAK | 1 ~ VERY STRONG  2 ~ SOMEWHAT STRONG  3 ~ SOMEWHAT WEAK  4 ~ VERY WEAK |
| Social Factors | Coping - social support  (STS_4) | When faced with this source of stress (Author’s Note – this references Greatest Source of Stress / STS_3), you can count on people that you know to help you deal with the situation. Do you...? | 1 ~ STRONGLY AGREE  2 ~ AGREE  3 ~ NEITHER AGREE NOR DISAGREE  4 ~ DISAGREE  5 ~ STRONGLY DISAGREE | 1 ~ STRONGLY AGREE  2 ~ AGREE  3 ~ NEITHER AGREE NOR DISAGREE  4 ~ DISAGREE  5 ~ STRONGLY DISAGREE |
| Social Factors | Difficulty in community activities  (DAS_04) | In the last 30 days, how much of a problem did you have joining in community activities (for example, festivities, religious or other activities) in the same way as anyone else can? | 1 ~ NONE  2 ~ MILD  3 ~ MODERATE  4 ~ SEVERE  5 ~ EXTREME/CANNOT DO | 1 ~ NONE  2 ~ MILD  3 ~ MODERATE  4 ~ SEVERE  5 ~ EXTREME/CANNOT DO |
| Social Factors | Difficulty maintaining friendship  (DAS_11) | In the last 30 days, how much difficulty did you have in maintaining a friendship? | 1 ~ NONE  2 ~ MILD  3 ~ MODERATE  4 ~ SEVERE  5 ~ EXTREME/CANNOT DO | 1 ~ NONE  2 ~ MILD  3 ~ MODERATE  4 ~ SEVERE  5 ~ EXTREME/CANNOT DO |
| Social Factors | Difficulty with new people  (DAS_10) | In the last 30 days, how much difficulty did you have in dealing with people you do not know? | 1 ~ NONE  2 ~ MILD  3 ~ MODERATE  4 ~ SEVERE  5 ~ EXTREME/CANNOT DO | 1 ~ NONE  2 ~ MILD  3 ~ MODERATE  4 ~ SEVERE  5 ~ EXTREME/CANNOT DO |
| Social Factors | Negative social interactions  (NSIDSC) | Negative social interactions - scale | 0 – 12 ~ SCORE | 0 – 12 ~ SCORE |
| Social Factors | Social provisions  (SPSDCON) | Social Provisions Scale - overall score | 10 – 40 ~ SCORE | [Score reverse coded] |
| Outcome | Psychological Stress  (GEN_07) | Thinking about the amount of stress in your life, would you say that most days are...? | 1 ~ NOT AT ALL STRESSFUL  2 ~ NOT VERY STRESSFUL  3 ~ A BIT STRESSFUL  4 ~ QUITE A BIT STRESSFUL  5 ~ EXTREMELY STRESSFUL | 1 ~ NOT AT ALL STRESSFUL  2 ~ NOT VERY STRESSFUL  3 ~ A BIT STRESSFUL  4 ~ QUITE A BIT STRESSFUL  5 ~ EXTREMELY STRESSFUL |
