# Supplementary material for: Multidimensional correlates of psychological stress: Insights from traditional statistical approaches and machine learning using a nationally representative Canadian sample
Source: PLoS One. 2025 May 13;20(5):e0323197. doi: 10.1371/journal.pone.0323197 (PMC12074393; doi:10.1371/journal.pone.0323197)
Supplement: S2 Table — (DOCX) [file pone.0323197.s002.docx]

***Table S2 –*** ***Weighted Sample Demographic Information***

| **Variable** | **Weighted** |
| --- | --- |
| **Sex**, % Female | 51% |
|  |  |
| **Marital Status** |  |
| Married | 54% |
| Common law | 12% |
| Widow | 5% |
| Divorced | 9% |
| Single | 21% |
| Not stated | <1% |
|  |  |
| **Minority Status** |  |
| White | 77% |
| Non-White | 22% |
| Not Stated | <1% |
|  |  |
| **Education** |  |
| Less than Secondary | 15% |
| Secondary School | 16% |
| Other Post-Secondary | 6% |
| Post-secondary degree | 63% |
| Not Stated | 1% |
|  |  |
| **Age** |  |
| 20-24 | 8% |
| 25-29 | 9% |
| 30-34 | 8% |
| 35-39 | 9% |
| 40-44 | 10% |
| 45-49 | 9% |
| 50-54 | 11% |
| 55-59 | 9% |
| 60-64 | 8% |
| 65-69 | 7% |
| 70-75 | 5% |
| 75-79 | 3% |
| 80+ | 4% |
|  |  |
| **Province** |  |
| British Columbia | 14% |
| Alberta | 11% |
| Saskatchewan | 3% |
| Manitoba | 3% |
| Ontario | 39% |
| Quebec | 24% |
| New Brunswick | 2% |
| Nova Scotia | 3% |
| PEI | <1% |
| Newfoundland & Labrador | 2% |
