# Supplementary material for: Multidimensional correlates of psychological stress: Insights from traditional statistical approaches and machine learning using a nationally representative Canadian sample
Source: PLoS One. 2025 May 13;20(5):e0323197. doi: 10.1371/journal.pone.0323197 (PMC12074393; doi:10.1371/journal.pone.0323197)
Supplement: S3 Table — Missingness of each variable and primary random forest analysis, in terms of both raw (Imp.) and relative (Rel. Imp.) importance. (DOCX) [file pone.0323197.s003.docx]

***Table S3 - Missingness of Data and Complete Analysis Results.*** *Missingness of each variable and primary random forest analysis, in terms of both raw (Imp.) and relative (Rel. Imp.) importance.*

| **Category** | **Name** | **Missing (%)** | **Random Forest Model** | |
| --- | --- | --- | --- | --- |
|  |  |  | **Imp.** | **Rel. Imp.** |
| Psychological Factors | Life Satisfaction (R) | 0.45% | 0.103 | 1.000 (1) |
| Social Factors | Negative Social Interactions | 1.23% | 0.102 | 0.991 (2) |
| Psychological Factors | Stress Source | 0.74% | 0.088 | 0.854 (3) |
| Demographics | Age (R) | 0.00% | 0.079 | 0.768 (4) |
| Demographics | Employment | 12.01% | 0.037 | 0.355 (5) |
| Psychological Factors | Emotional Impact of Health | 0.32% | 0.025 | 0.244 (6) |
| Health Behaviour | Level of Insomnia | 0.09% | 0.025 | 0.240 (7) |
| Physical Health | Self-Perceived Health | 0.03% | 0.013 | 0.127 (8) |
| Social Factors | Coping – Social Support | 15.93% | 0.01 | 0.099 (9) |
| Demographics | Household Type | 0.32% | 0.009 | 0.089 (10) |
| Psychological Factors | Coping on a Daily Basis | 0.41% | 0.009 | 0.086 (11) |
| Social Factors | SPS - Total Score (R) | 3.63% | 0.007 | 0.072 (12) |
| Psychological Factors | Coping with Crisis | 0.29% | 0.007 | 0.071 (13) |
| Demographics | Household Size | 0.02% | 0.007 | 0.070 (14) |
| Demographics | Income (Provincial) (R) | 0.04% | 0.007 | 0.068 (15) |
| Demographics | Marital Status | 0.25% | 0.006 | 0.059 (16) |
| Physical Health | Difficulty Walking | 0.62% | 0.006 | 0.057 (17) |
| Demographics | Education | 0.46% | 0.005 | 0.053 (18) |
| Physical Health | Difficulty Household Responsibilities | 0.27% | 0.005 | 0.050 (19) |
| Psychological Factors | Coping Skill | 15.88% | 0.005 | 0.050 (20) |
| Mental Health | Generalized Anxiety Disorder | 0.95% | 0.005 | 0.045 (21) |
| Mental Health | Major Depression | 0.66% | 0.005 | 0.044 (22) |
| Demographics | Body Mass Index | 3.38% | 0.005 | 0.044 (23) |
| Health Behaviour | Frequency of Drinking | 0.13% | 0.004 | 0.043 (24) |
| Physical Health | Difficulty Standing | 0.20% | 0.004 | 0.040 (25) |
| Demographics | Sex | 0.00% | 0.004 | 0.040 (26) |
| Psychological Factors | Difficulty Concentrating | 0.32% | 0.004 | 0.039 (27) |
| Physical Health | High Blood Pressure | 0.15% | 0.004 | 0.038 (28) |
| Physical Health | Arthritis | 0.09% | 0.004 | 0.035 (29) |
| Mental Health | Anxiety Disorder | 0.04% | 0.003 | 0.034 (30) |
| Physical Health | Back Problems | 0.04% | 0.003 | 0.033 (31) |
| Life Adversity | ELA - Sum Score | 2.28% | 0.003 | 0.029 (32) |
| Health Behaviour | Weekly hours of MVPA | 0.25% | 0.003 | 0.028 (33) |
| Social Factors | Community Belonging | 0.75% | 0.003 | 0.027 (34) |
| Life Adversity | RLA – Unmet needs | 0.54% | 0.003 | 0.027 (35) |
| Demographics | Province | 0.00% | 0.003 | 0.027 (36) |
| Physical Health | Migraines | 0.05% | 0.002 | 0.023 (37) |
| Health Behaviour | Smoking Status | 0.13% | 0.002 | 0.023 (38) |
| Demographics | Dwelling Type | 0.02% | 0.002 | 0.022 (39) |
| Mental Health | Suicidal Thoughts | 0.29% | 0.002 | 0.019 (40) |
| Physical Health | Heart Disease | 0.12% | 0.002 | 0.019 (41) |
| Social Factors | Difficulty In Community Activities | 7.27% | 0.002 | 0.016 (42) |
| Social Factors | Difficulty with New People | 0.30% | 0.001 | 0.013 (43) |
| Demographics | Minority Status | 0.41% | 0.001 | 0.012 (44) |
| Life Adversity | RLA - Witness a Crime | 0.19% | 0.001 | 0.012 (45) |
| Demographics | Student Status | 0.32% | 0.001 | 0.011 (46) |
| Physical Health | Diabetes | 0.03% | 0.001 | 0.011 (47) |
| Physical Health | Chronic Fatigue | 0.08% | 0.001 | 0.010 (48) |
| Physical Health | Previous Cancer | 2.47% | 0.001 | 0.008 (49) |
| Physical Health | Bowel Disorders | 0.07% | 0.001 | 0.006 (50) |
| Social Factors | Difficulty Maintaining Friendship | 0.33% | 0.001 | 0.006 (51) |
| Health Behaviour | Illicit Drug Use | 1.02% | 0.001 | 0.005 (52) |
| Life Adversity | RLA – Family Problems | 0.20% | 0.001 | 0.005 (53) |
| Demographics | Immigrant Status | 0.58% | 0.000 | 0.005 (54) |
| Mental Health | Bipolar Disorder | 0.59% | 0.000 | 0.004 (55) |
| Mental Health | Learning Disability | 0.08% | 0.000 | 0.003 (56) |
| Physical Health | Asthma | 0.04% | 0.000 | 0.003 (57) |
| Mental Health | Attention Deficit Disorder | 0.07% | 0.000 | 0.003 (58) |
| Life Adversity | RLA - Victim of a Crime | 0.19% | 0.000 | 0.003 (59) |
| Mental Health | PSTD | 0.10% | 0.000 | 0.002 (60) |
| Health Behaviour | WHO alcohol abuse or dependence | 1.52% | 0.000 | 0.001 (61) |
| Mental Health | Mania | 0.50% | 0.000 | 0.000 (62) |
| Physical Health | Current Cancer | 0.06% | 0.000 | 0.000 (63) |
| Health Behaviour | WHO drug abuse or dependence | 1.52% | 0.000 | 0.000 (64) |
| Mental Health | Hypomanic | 0.54% | 0.000 | 0.000 (65) |
| Physical Health | Stroke | 0.06% | 0.000 | 0.000 (66) |

(R) denotes reverse-coded variables
Note: Negative relative importance represents a small negative raw importance which was rounded to 0
