# Supplementary material for: Multidimensional correlates of psychological stress: Insights from traditional statistical approaches and machine learning using a nationally representative Canadian sample
Source: PLoS One. 2025 May 13;20(5):e0323197. doi: 10.1371/journal.pone.0323197 (PMC12074393; doi:10.1371/journal.pone.0323197)
Supplement: S4 Table — The raw importance (Imp), relative importance (RI), and rank within each analysis (Rank) of each sensitivity analysis. Variables are listed in the order of importance in the original analysis with the complete dataset; rank denotes the level of importance in each sensitivity analysis. The Age (20–75) analysis includes only those between the ages of 20 and 75; this omits those over age 75, who were not asked the employment question. The No Coping analysis omitted two coping items (i.e., Coping - social support, coping skill), which were not asked of those who reported no primary stressor. Finally, the No Mental Illness analysis omits those who reported having mental illness including: depression, bipolar disorder, any anxiety disorder, PTSD, suicidal thoughts, mania, and hypomania. Negative social interactions, life satisfaction (R), and Age (R) were consistently seen to be three of the most important variables. (DOCX) [file pone.0323197.s004.docx]

***Table S4 -*** ***Sensitivity Analyses.*** *The raw importance (Imp), relative importance (RI), and rank within each analysis (Rank) of each sensitivity analysis. Variables are listed in the order of importance in the original analysis with the complete dataset; rank denotes the level of importance in each sensitivity analysis. The Age (20-75) analysis includes only those between the ages of 20 and 75; this omits those over age 75, who were not asked the employment question. The No Coping analysis omitted two coping items (i.e., Coping - social support, coping skill), which were not asked of those who reported no primary stressor. Finally, the No Mental Illness analysis omits those who reported having mental illness including: depression, bipolar disorder, any anxiety disorder, PTSD, suicidal thoughts, mania, and hypomania. Negative social interactions, life satisfaction (R), and age (R) were consistently seen to be three of the most important variables.*

|  | **Primary Analysis**  **(N = 23,089)** | | **Age (20-75)**  **(N = 22,346)** | | | | **No Coping  (N = 23,089)** | | **No Mental Illness**  **(N = 19,775)** | |
| --- | --- | --- | --- | --- | --- | --- | --- | --- | --- | --- |
| **Lay Name** | **Imp.** | **RI (Rank)** | | **Imp.** | **RI (Rank)** | **Imp.** | | **RI (Rank)** | **Imp.** | **RI (Rank)** |
| Life Satisfaction (R) | 0.103 | 1.000 (1) | | 0.111 | 1.000 (1) | 0.109 | | 1.000 (1) | 0.068 | 0.724 (4) |
| Negative Social Interactions | 0.102 | 0.991 (2) | | 0.096 | 0.863 (2) | 0.09 | | 0.826 (2) | 0.072 | 0.764 (3) |
| Stress Source | 0.088 | 0.854 (3) | | 0.081 | 0.730 (3) | 0.075 | | 0.692 (3) | 0.094 | 1.000 (1) |
| Age (R) | 0.079 | 0.768 (4) | | 0.056 | 0.502 (4) | 0.054 | | 0.491 (4) | 0.075 | 0.804 (2) |
| Employment | 0.037 | 0.355 (5) | | 0.027 | 0.240 (5) | 0.028 | | 0.253 (5) | 0.034 | 0.358 (5) |
| Emotional Impact of Health | 0.025 | 0.244 (6) | | 0.018 | 0.161 (7) | 0.02 | | 0.181 (7) | 0.010 | 0.102 (10) |
| Level of Insomnia | 0.025 | 0.240 (7) | | 0.025 | 0.224 (6) | 0.025 | | 0.230 (6) | 0.023 | 0.245 (6) |
| Self-Perceived Health | 0.013 | 0.127 (8) | | 0.012 | 0.104 (9) | 0.012 | | 0.111 (8) | 0.009 | 0.092 (11) |
| Coping – Social Support | 0.010 | 0.099 (9) | | 0.013 | 0.115 (8) |  | |  | 0.013 | 0.139 (7) |
| Household Type | 0.009 | 0.089 (10) | | 0.009 | 0.080 (12) | 0.01 | | 0.096 (9) | 0.012 | 0.128 (8) |
| Coping on a Daily Basis | 0.009 | 0.086 (11) | | 0.009 | 0.083 (10) | 0.01 | | 0.088 (10) | 0.007 | 0.078 (13) |
| SPS - Total Score (R) | 0.007 | 0.072 (12) | | 0.009 | 0.080 (11) | 0.007 | | 0.069 (14) | 0.007 | 0.075 (14) |
| Coping with Crisis | 0.007 | 0.071 (13) | | 0.008 | 0.069 (13) | 0.008 | | 0.071 (12) | 0.006 | 0.063 (16) |
| Household Size | 0.007 | 0.070 (14) | | 0.007 | 0.067 (14) | 0.008 | | 0.077 (11) | 0.010 | 0.102 (9) |
| Income (Provincial) (R) | 0.007 | 0.068 (15) | | 0.006 | 0.058 (15) | 0.008 | | 0.070 (13) | 0.006 | 0.062 (17) |
| Marital Status | 0.006 | 0.059 (16) | | 0.004 | 0.040 (24) | 0.005 | | 0.042 (20) | 0.007 | 0.080 (12) |
| Difficulty Walking | 0.006 | 0.057 (17) | | 0.004 | 0.034 (26) | 0.005 | | 0.048 (18) | 0.005 | 0.049 (21) |
| Education | 0.005 | 0.053 (18) | | 0.005 | 0.041 (22) | 0.004 | | 0.040 (22) | 0.006 | 0.067 (15) |
| Difficulty Household Responsibilities | 0.005 | 0.050 (19) | | 0.006 | 0.051 (18) | 0.006 | | 0.059 (16) | 0.003 | 0.029 (27) |
| Coping Skill | 0.005 | 0.050 (20) | | 0.006 | 0.055 (16) |  | |  | 0.005 | 0.050 (20) |
| Generalized Anxiety Disorder | 0.005 | 0.045 (21) | | 0.003 | 0.028 (30) | 0.004 | | 0.035 (27) |  |  |
| Major Depression | 0.005 | 0.044 (22) | | 0.006 | 0.054 (17) | 0.006 | | 0.059 (15) |  |  |
| Body Mass Index | 0.005 | 0.044 (23) | | 0.005 | 0.043 (20) | 0.004 | | 0.039 (23) | 0.002 | 0.022 (29) |
| Frequency of Drinking | 0.004 | 0.043 (24) | | 0.005 | 0.041 (23) | 0.004 | | 0.037 (25) | 0.005 | 0.052 (19) |
| Difficulty Standing | 0.004 | 0.040 (25) | | 0.004 | 0.032 (29) | 0.004 | | 0.038 (24) | 0.004 | 0.043 (23) |
| Sex | 0.004 | 0.040 (26) | | 0.005 | 0.041 (21) | 0.004 | | 0.041 (21) | 0.003 | 0.032 (24) |
| Difficulty Concentrating | 0.004 | 0.039 (27) | | 0.005 | 0.047 (19) | 0.006 | | 0.056 (17) | 0.001 | 0.010 (37) |
| High Blood Pressure | 0.004 | 0.038 (28) | | 0.003 | 0.024 (35) | 0.003 | | 0.025 (32) | 0.005 | 0.053 (18) |
| Arthritis | 0.004 | 0.035 (29) | | 0.003 | 0.027 (32) | 0.004 | | 0.036 (26) | 0.004 | 0.045 (22) |
| Anxiety Disorder | 0.003 | 0.034 (30) | | 0.004 | 0.032 (28) | 0.003 | | 0.030 (30) |  |  |
| Back Problems | 0.003 | 0.033 (31) | | 0.003 | 0.024 (34) | 0.002 | | 0.020 (34) | 0.002 | 0.019 (31) |
| ELA - Sum Score | 0.003 | 0.029 (32) | | 0.004 | 0.035 (25) | 0.004 | | 0.034 (28) | 0.002 | 0.017 (33) |
| Weekly hours of MVPA | 0.003 | 0.028 (33) | | 0.002 | 0.021 (37) | 0.003 | | 0.029 (31) | 0.003 | 0.030 (25) |
| Community Belonging | 0.003 | 0.027 (34) | | 0.003 | 0.023 (36) | 0.002 | | 0.019 (35) | 0.002 | 0.020 (30) |
| RLA – Unmet needs | 0.003 | 0.027 (35) | | 0.003 | 0.028 (31) | 0.005 | | 0.042 (19) | 0.001 | 0.010 (38) |
| Province | 0.003 | 0.027 (36) | | 0.004 | 0.034 (27) | 0.003 | | 0.032 (29) | 0.003 | 0.029 (26) |
| Migraines | 0.002 | 0.023 (37) | | 0.002 | 0.016 (41) | 0.002 | | 0.016 (38) | 0.002 | 0.017 (34) |
| Smoking Status | 0.002 | 0.023 (38) | | 0.002 | 0.018 (39) | 0.002 | | 0.015 (41) | 0.002 | 0.017 (32) |
| Dwelling Type | 0.002 | 0.022 (39) | | 0.002 | 0.020 (38) | 0.002 | | 0.018 (36) | 0.002 | 0.026 (28) |
| Suicidal Thoughts | 0.002 | 0.019 (40) | | 0.003 | 0.026 (33) | 0.003 | | 0.024 (33) |  |  |
| Heart Disease | 0.002 | 0.019 (41) | | 0.000 | 0.004 (53) | 0.000 | | 0.004 (52) | 0.001 | 0.016 (35) |
| Difficulty In Community Activities | 0.002 | 0.016 (42) | | 0.002 | 0.014 (42) | 0.002 | | 0.016 (40) | 0.001 | 0.009 (41) |
| Difficulty with New People | 0.001 | 0.013 (43) | | 0.001 | 0.011 (43) | 0.001 | | 0.013 (42) | 0.000 | 0.001 (51) |
| Minority Status | 0.001 | 0.012 (44) | | 0.002 | 0.016 (40) | 0.002 | | 0.017 (37) | 0.001 | 0.014 (36) |
| RLA - Witness a Crime | 0.001 | 0.012 (45) | | 0.001 | 0.006 (51) | 0.001 | | 0.008 (49) | 0.000 | 0.001 (50) |
| Student Status | 0.001 | 0.011 (46) | | 0.001 | 0.010 (44) | 0.002 | | 0.016 (39) | 0.001 | 0.010 (39) |
| Diabetes | 0.001 | 0.011 (47) | | 0.001 | 0.008 (48) | 0.001 | | 0.009 (44) | 0.001 | 0.008 (42) |
| Chronic Fatigue | 0.001 | 0.010 (48) | | 0.001 | 0.007 (50) | 0.001 | | 0.008 (46) | 0.000 | 0.004 (46) |
| Previous Cancer | 0.001 | 0.008 (49) | | 0.000 | 0.004 (54) | 0.001 | | 0.007 (50) | 0.001 | 0.009 (40) |
| Bowel Disorders | 0.001 | 0.006 (50) | | 0.001 | 0.008 (47) | 0.001 | | 0.008 (45) | 0.000 | 0.003 (48) |
| Difficulty Maintaining Friendship | 0.001 | 0.006 (51) | | 0.001 | 0.008 (46) | 0.001 | | 0.010 (43) | 0.000 | -0.001 (54) |
| Illicit Drug Use | 0.001 | 0.005 (52) | | 0.001 | 0.007 (49) | 0.001 | | 0.008 (48) | 0.000 | 0.002 (49) |
| RLA – Family Problems | 0.001 | 0.005 (53) | | 0.000 | 0.002 (59) | 0.000 | | 0.002 (56) | 0.001 | 0.006 (44) |
| Immigrant Status | 0.000 | 0.005 (54) | | 0.001 | 0.010 (45) | 0.001 | | 0.008 (47) | 0.001 | 0.008 (43) |
| Bipolar Disorder | 0.000 | 0.004 (55) | | 0.000 | 0.004 (55) | 0.000 | | 0.004 (53) |  |  |
| Learning Disability | 0.000 | 0.003 (56) | | 0.000 | 0.000 (60) | 0.000 | | 0.002 (57) | 0.000 | 0.001 (52) |
| Asthma | 0.000 | 0.003 (57) | | 0.001 | 0.006 (52) | 0.001 | | 0.005 (51) | 0.000 | 0.004 (45) |
| Attention Deficit Disorder | 0.000 | 0.003 (58) | | 0.000 | 0.000 (61) | 0.000 | | 0.001 (58) | 0.000 | 0.000 (53) |
| RLA - Victim of a Crime | 0.000 | 0.003 (59) | | 0.000 | 0.004 (56) | 0.000 | | 0.003 (54) | 0.000 | 0.003 (47) |
| PSTD | 0.000 | 0.002 (60) | | 0.000 | 0.002 (58) | 0.000 | | 0.001 (59) |  |  |
| WHO alcohol abuse or dependence | 0.000 | 0.001 (61) | | 0.000 | 0.003 (57) | 0.000 | | 0.002 (55) | 0.000 | -0.001 (55) |
| Mania | 0.000 | 0.000 (62) | | 0.000 | -0.001 (64) | 0.000 | | 0.000 (61) |  |  |
| Current Cancer | 0.000 | 0.000 (63) | | 0.000 | -0.001 (65) | 0.000 | | 0.000 (62) | 0.000 | -0.001 (56) |
| WHO drug abuse or dependence | 0.000 | 0.000 (64) | | 0.000 | 0.000 (63) | 0.000 | | 0.000 (60) | 0.000 | -0.001 (57) |
| Hypomanic | 0.000 | 0.000 (65) | | 0.000 | 0.000 (62) | 0.000 | | 0.000 (63) |  |  |
| Stroke | 0.000 | -0.001 (66) | | 0.000 | -0.002 (66) | 0.000 | | -0.002 (64) | 00.000 | -0.003 (58) |

(R) denotes reverse-coded variables
