# Supplementary figures and images for: Multidimensional correlates of psychological stress: Insights from traditional statistical approaches and machine learning using a nationally representative Canadian sample
Source: PLoS One. 2025 May 13;20(5):e0323197. doi: 10.1371/journal.pone.0323197 (PMC12074393; doi:10.1371/journal.pone.0323197)

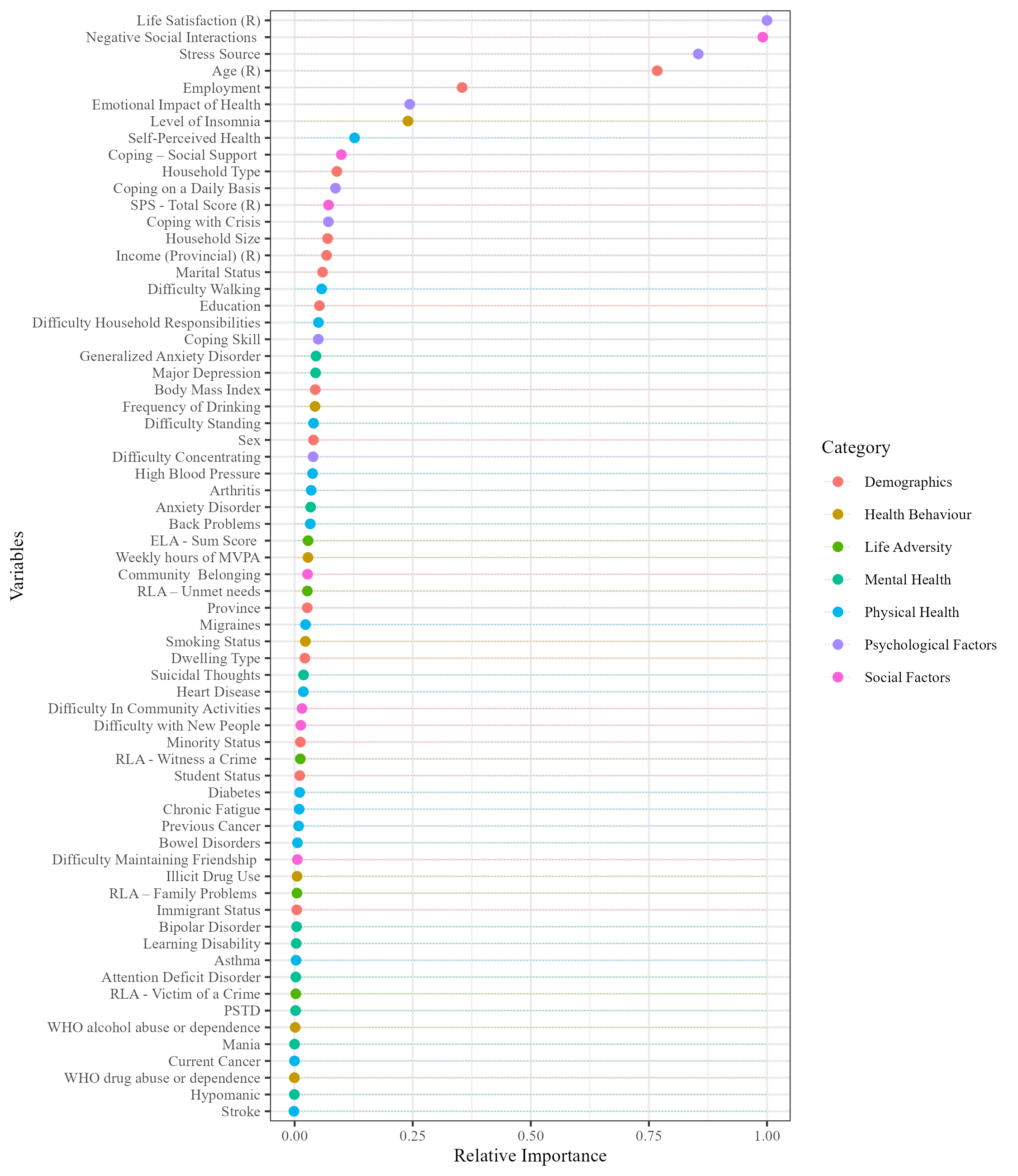

Supplement: S1 Figure — (R) denotes variables that are reverse coded. (TIFF) [file pone.0323197.s005.tiff]
