# Supplementary material for: Multidimensional correlates of psychological stress: Insights from traditional statistical approaches and machine learning using a nationally representative Canadian sample
Source: PLoS One. 2025 May 13;20(5):e0323197. doi: 10.1371/journal.pone.0323197 (PMC12074393; doi:10.1371/journal.pone.0323197)
Supplement: S1 File — (DOCX) [file pone.0323197.s006.docx]

**Regression Results**

**Multiple Linear Regression**

Call:

lm(formula = Stress ~ life_disatisfaction + neg_social_interaction +

age_R + stress_source + prev_yr_employment, data = linear.regression.data,

weights = WTS_M)

Weighted Residuals:

Min 1Q Median 3Q Max

-273.62 -14.46 0.42 12.92 399.16

Coefficients:

Estimate Std. Error t value Pr(>|t|)

(Intercept) -0.70388 0.018685 -37.671 < 2e-16 ***

life_disatisfaction 0.235336 0.006296 37.381 < 2e-16 ***

neg_social_interaction 0.213149 0.006134 34.749 < 2e-16 ***

age_R 0.034416 0.008338 4.128 3.68e-05 ***

stress_sourceCARING FOR CHILD 0.718990 0.036194 19.865 < 2e-16 ***

stress_sourceCARING FOR OTHER 0.756401 0.055502 13.628 < 2e-16 ***

stress_sourceDISCRIMINATION 0.520642 0.129773 4.012 6.04e-05 ***

stress_sourceEMPLOYMNT STATUS 0.720210 0.039941 18.032 < 2e-16 ***

stress_sourceFINANCIAL SIT. 0.702844 0.022785 30.847 < 2e-16 ***

stress_sourceHEALTH OF FAMILY 0.672786 0.027203 24.732 < 2e-16 ***

stress_sourceLOSS OF LOV. ONE 0.667900 0.118613 5.631 1.81e-08 ***

stress_sourceMENTAL HEALTH 0.773418 0.040553 19.072 < 2e-16 ***

stress_sourceOTHER 0.508667 0.032238 15.779 < 2e-16 ***

stress_sourcePERS. RELATIONS. 0.656439 0.030354 21.626 < 2e-16 ***

stress_sourcePERS./FAM. RESP. 0.662203 0.028800 22.993 < 2e-16 ***

stress_sourcePERSONAL SAFETY 0.633496 0.042771 14.812 < 2e-16 ***

stress_sourcePHYSICAL HEALTH 0.633765 0.027947 22.677 < 2e-16 ***

stress_sourceSCHOOL 0.782165 0.041219 18.976 < 2e-16 ***

stress_sourceTIME PRESSURE 0.766740 0.025645 29.898 < 2e-16 ***

stress_sourceWORK. SITUATION 0.838329 0.021625 38.766 < 2e-16 ***

prev_yr_employment 0.197685 0.015486 12.765 < 2e-16 ***

---

Signif. codes: 0 ‘***’ 0.001 ‘**’ 0.01 ‘*’ 0.05 ‘.’ 0.1 ‘ ’ 1

Residual standard error: 28.74 on 23068 degrees of freedom

Multiple R-squared: 0.265, Adjusted R-squared: 0.2644

F-statistic: 415.9 on 20 and 23068 DF, p-value: < 2.2e-16

**Life Satisfaction**

Call:

lm(formula = Stress ~ life_disatisfaction, data = linear.regression.data,

weights = WTS_M)

Weighted Residuals:

Min 1Q Median 3Q Max

-267.27 -17.97 2.13 13.54 406.31

Coefficients:

Estimate Std. Error t value Pr(>|t|)

(Intercept) 0.075754 0.006259 12.10 <2e-16 ***

life_disatisfaction 0.316714 0.006536 48.46 <2e-16 ***

---

Signif. codes: 0 ‘***’ 0.001 ‘**’ 0.01 ‘*’ 0.05 ‘.’ 0.1 ‘ ’ 1

Residual standard error: 31.92 on 23087 degrees of freedom

Multiple R-squared: 0.09232, Adjusted R-squared: 0.09228

F-statistic: 2348 on 1 and 23087 DF, p-value: < 2.2e-16

**Negative Social Interaction**

Call:

lm(formula = Stress ~ neg_social_interaction, data = linear.regression.data,

weights = WTS_M)

Weighted Residuals:

Min 1Q Median 3Q Max

-277.42 -16.87 1.47 14.33 422.39

Coefficients:

Estimate Std. Error t value Pr(>|t|)

(Intercept) 0.060957 0.006137 9.933 <2e-16 ***

neg_social_interaction 0.356125 0.006126 58.138 <2e-16 ***

---

Signif. codes: 0 ‘***’ 0.001 ‘**’ 0.01 ‘*’ 0.05 ‘.’ 0.1 ‘ ’ 1

Residual standard error: 31.29 on 23087 degrees of freedom

Multiple R-squared: 0.1277, Adjusted R-squared: 0.1277

F-statistic: 3380 on 1 and 23087 DF, p-value: < 2.2e-16

**Age**

Call:

lm(formula = Stress ~ age_R, data = linear.regression.data, weights = WTS_M)

Weighted Residuals:

Min 1Q Median 3Q Max

-308.98 -17.95 2.16 14.15 420.87

Coefficients:

Estimate Std. Error t value Pr(>|t|)

(Intercept) 0.066785 0.006432 10.38 <2e-16 ***

age_R 0.237618 0.007541 31.51 <2e-16 ***

---

Signif. codes: 0 ‘***’ 0.001 ‘**’ 0.01 ‘*’ 0.05 ‘.’ 0.1 ‘ ’ 1

Residual standard error: 32.81 on 23087 degrees of freedom

Multiple R-squared: 0.04124, Adjusted R-squared: 0.0412

F-statistic: 993 on 1 and 23087 DF, p-value: < 2.2e-16

**Level of Insomnia**

Call:

lm(formula = Stress ~ sleep_trouble, data = linear.regression.data,

weights = WTS_M)

Weighted Residuals:

Min 1Q Median 3Q Max

-322.06 -19.29 2.24 14.08 406.11

Coefficients:

Estimate Std. Error t value Pr(>|t|)

(Intercept) 0.084686 0.006418 13.20 <2e-16 ***

sleep_trouble 0.229441 0.006608 34.72 <2e-16 ***

---

Signif. codes: 0 ‘***’ 0.001 ‘**’ 0.01 ‘*’ 0.05 ‘.’ 0.1 ‘ ’ 1

Residual standard error: 32.66 on 23087 degrees of freedom

Multiple R-squared: 0.04963, Adjusted R-squared: 0.04959

F-statistic: 1206 on 1 and 23087 DF, p-value: < 2.2e-16

**Employment**

Call:

lm(formula = Stress ~ prev_yr_employment, data = linear.regression.data,

weights = WTS_M)

Weighted Residuals:

Min 1Q Median 3Q Max

-264.34 -17.60 3.03 13.83 409.97

Coefficients:

Estimate Std. Error t value Pr(>|t|)

(Intercept) -0.25197 0.01197 -21.05 <2e-16 ***

prev_yr_employment 0.45193 0.01419 31.85 <2e-16 ***

---

Signif. codes: 0 ‘***’ 0.001 ‘**’ 0.01 ‘*’ 0.05 ‘.’ 0.1 ‘ ’ 1

Residual standard error: 32.79 on 23087 degrees of freedom

Multiple R-squared: 0.04208, Adjusted R-squared: 0.04204

F-statistic: 1014 on 1 and 23087 DF, p-value: < 2.2e-16

**Stress Source**

Call:

lm(formula = Stress ~ stress_source, data = linear.regression.data,

weights = WTS_M)

Weighted Residuals:

Min 1Q Median 3Q Max

-274.65 -17.02 1.80 10.68 407.02

Coefficients:

Estimate Std. Error t value Pr(>|t|)

(Intercept) -0.85387 0.01695 -50.390 < 2e-16 ***

stress_sourceCARING FOR CHILD 1.01740 0.03843 26.474 < 2e-16 ***

stress_sourceCARING FOR OTHER 1.06809 0.05957 17.929 < 2e-16 ***

stress_sourceDISCRIMINATION 0.80865 0.13998 5.777 7.71e-09 ***

stress_sourceEMPLOYMNT STATUS 1.06913 0.04225 25.307 < 2e-16 ***

stress_sourceFINANCIAL SIT. 1.10561 0.02323 47.596 < 2e-16 ***

stress_sourceHEALTH OF FAMILY 0.86980 0.02912 29.870 < 2e-16 ***

stress_sourceLOSS OF LOV. ONE 0.86096 0.12796 6.729 1.75e-11 ***

stress_sourceMENTAL HEALTH 1.23759 0.04293 28.827 < 2e-16 ***

stress_sourceOTHER 0.75243 0.03453 21.790 < 2e-16 ***

stress_sourcePERS. RELATIONS. 1.12587 0.03165 35.578 < 2e-16 ***

stress_sourcePERS./FAM. RESP. 0.94976 0.03062 31.013 < 2e-16 ***

stress_sourcePERSONAL SAFETY 0.79365 0.04608 17.225 < 2e-16 ***

stress_sourcePHYSICAL HEALTH 0.89854 0.02956 30.397 < 2e-16 ***

stress_sourceSCHOOL 1.10740 0.04238 26.129 < 2e-16 ***

stress_sourceTIME PRESSURE 1.06916 0.02655 40.276 < 2e-16 ***

stress_sourceWORK. SITUATION 1.20979 0.02138 56.590 < 2e-16 ***

---

Signif. codes: 0 ‘***’ 0.001 ‘**’ 0.01 ‘*’ 0.05 ‘.’ 0.1 ‘ ’ 1

Residual standard error: 31.02 on 23072 degrees of freedom

Multiple R-squared: 0.1432, Adjusted R-squared: 0.1426

F-statistic: 241 on 16 and 23072 DF, p-value: < 2.2e-16
