# Supplementary material for: Multidimensional correlates of psychological stress: Insights from traditional statistical approaches and machine learning using a nationally representative Canadian sample
Source: PLoS One. 2025 May 13;20(5):e0323197. doi: 10.1371/journal.pone.0323197 (PMC12074393; doi:10.1371/journal.pone.0323197)
Supplement: Attachment — Submitted filename: Multidimensional Correlates - Revisions response letter.docx [file pone.0323197.s008.docx]

Dear Dr. Mahmoud,

Thank you for the opportunity to submit a revision to the manuscript (No. PONE-D-24-46724), “Multidimensional correlates of psychological stress: Insights from traditional statistical approaches and machine learning using a nationally representative Canadian sample.”

We thank you and the reviewer for the comments; they have strengthened the entirety of the submission. Below, we outline each comment from yourself and the reviewer and highlight how we have addressed them, including the page number to view the revisions to the text (all changes in the text are also highlighted using red font). Again, thank you for the opportunity to resubmit the manuscript for publication in *PLOS ONE*.

**Benjamin A. Hives, MSc** (he/him)
PhD Candidate, University of British Columbia

Faculty of Education | School of Kinesiology
The University of British Columbia | Vancouver Campus | Musqueam Traditional Territory
210-6081 University Blvd | Vancouver BC | V6T1Z1 Canada

**Reviewer 1**

­­­­­­­­­­­

***R1.1*** *The manuscript explores the correlates of psychological stress using both traditional statistical methods and a random forest algorithm. While it tackles an important public health issue and uses innovative methods, there are significant concerns about the methodology, particularly the way the random forest model was applied and evaluated.*

***R1.1 Response.*** Thank you for your thoughtful comments. We have addressed them in the text and provide descriptions of the changes below.

***R1.2.*** *The study uses random forest as its main machine-learning method but doesn’t follow common practices in the field. For example, the dataset wasn’t split into training, validation, and testing subsets. On top of that, there’s no mention of cross-validation, which is crucial for ensuring that the model is reliable and generalizable. Cross-validation methods, like k-fold or leave-one-out, are standard in machine learning to prevent overfitting and provide accurate error estimates. Without these steps, it’s hard to have confidence in the model’s performance. Adding these procedures in future revisions would greatly improve the robustness of the results.*

***R1.2 Response.*** Thank you for bringing this to our attention. We have shifted the syntax from basic ranger function to a *tidymodels* framework (packages included are listed p. 11, lines 201-204), allowing more cross-validation options. We have now included an explicit training/testing split rather than rely on OOB estimates provided by *ranger.* Additionally, we have included 10-fold cross-validation and hyperparmeter tuning (min node size, mtry) in the training set. These methods are highlighted in the Methods section (p. 8-9, lines 141-159):

The primary analysis for this study was random forest analysis [21], a decision tree-based machine learning method. Consistent with standard machine learning practices, the dataset was split into two subsets, a training set (75% of observations) for model development and a testing set (25% of observations) for evaluating the model’s performance with new data. To ensure similar distributions of the outcome variable in both datasets, stratified sampling (strata) was applied at this initial data splitting, which segmented the data of the outcome variable into quantiles and randomly sampled from each. To further assess model performance, we employed 10-fold cross-validation to evaluate the performance of a Random Forest model by partitioning the dataset into ten equal subsets, or 'folds'. In each iteration, the model was trained on nine folds and validated on the remaining fold, ensuring that every observation was used for both training and validation exactly once. This process helps assess the model’s generalizability and reduces the risk of overfitting.

Additionally, we performed hyperparameter tuning, using an automated search of 11 different hyperparameter combinations, including the number of variables tested at each split and the minimum number of observations required in a terminal node. The values for these two hyperparameters were automatically sampled from reasonable default ranges defined by the R package used in the analysis. The optimal model was selected based on the lowest mean squared error (MSE) obtained from cross-validation. Hyperparameter tuning helps further optimize model performance by improving predictive accuracy and reducing error rates and enhances generalization by increasing the model’s ability to perform well on new or unseen data.

Additional information was provided in the Results section (pp. 11-12, lines 216-218):

The random forest model included 1000 trees, as suggested by Breiman for analysis interested in variable importance[34]. Our final model included 21 variables at each split and had a minimum node size of ten.

While the results are fairly consistent with our previous findings (updated variable importance p. 12, lines 220-222), we agree with the reviewer that this provides robustness to the results.

***R1.3.*** *The manuscript also doesn’t include key metrics for evaluating the random forest model, such as out-of-bag error, mean squared error (MSE), root mean squared error (RMSE), or mean absolute error (MAE). These metrics are important for assessing how well the model performs and for supporting the choice of predictors in the follow-up analyses. It would also help to clarify if the variable importance measures came from out-of-bag samples, which is a standard and reliable practice for random forest models.*

***R1.3 Response.*** Mean squared error (along with R^2^) was previously reported for both the primary analysis and sensitivity analyses in Table 4. However, we have expanded on this in several sections by providing additional context and including RMSE and MAE. In the Sensitivity Analysis section (pp. 10-11, lines 186-195), we describe each term and their use in evaluating the models.

To compare these analyses to the primary analysis, we used three common error measures (i.e., MSE, root mean squared error, mean absolute error) and one effect size (i.e., R_2_)[24]. MSE is the average of squared differences between predicted and actual data, while Root mean squared error (RMSE), the square root of the MSE, provides an error measure on the same scale as the original data (i.e., stress score). Mean absolute error (MAE) is the average of the absolute differences between predicted and actual data, which is less influenced by outliers. R2 represents the proportion of the variance in the outcome variables explained by the model, assessing an overall model fit. Consistency between these sensitivity analyses and the primary analysis would confirm the robustness of the findings, demonstrating that the results were not unduly influenced by missing data or the inclusion of participants with mental illness.

Finally, the original table has been expanded to also include RMSE and MAE for all models (pp. 15, lines 274-275).

|  | **Primary Model**  **(N = 23,089)** | **Age (20-75)**  **(N = 22,346)** | **No Coping  (N = 21,171)** | **No Mental Illness**  **(N = 19,775)** |
| --- | --- | --- | --- | --- |
| ***MSE*** | 0.700 | 0.695 | 0.701 | 0.688 |
| ***RMSE*** | 0.837 | 0.834 | 0.837 | 0.829 |
| ***MAE*** | 0.664 | 0.656 | 0.665 | 0.660 |
| ***R^2^*** | 0.31 | 0.29 | 0.31 | 0.29 |

MSE, mean squared error; RMSE, root mean squared error; MAE, mean absolute error.

***R1.4.*** *Another concern is the use of a single-item measure for psychological stress. Treating this as a continuous variable without enough evidence to support its validity might affect the modeling outcomes. This limitation should be addressed in the discussion section.*

***R1.4 Response.*** While we had previously mentioned this in the discussion, we have added further details to provide the reader with additional context (pp. 18-19, lines 345-359).

Finally, there are two limitations regarding our measure of psychological stress (i.e., the Statistics Canada single-item stress measure). First, the measure of psychological stress used by Statistics Canada has not been validated. While it has been used by Statistics Canada and other researchers using Statistics Canada data [44–46] as a measure of psychological stress, future validation research is required to ensure that this measure is assessing an individual’s levels of psychological stress, rather than other mental ill-being measures. The second limitation of the single-item measure is the response scale, which is a 5-point Likert-type response scale. Treating Likert-type responses as continuous data is a contentious issue, with some researchers contending it should be treated as ordinal and others contending it can be treated as continuous [51]. Given the robustness of the analyses used (i.e., linear regression models, random forest algorithm), as well as the approximately normal distribution of the responses to the psychology stress item, we decided to treat it as continuous. However, future research should attempt to replicate this study using a measure of psychological stress (e.g., Perceived Stress Scale [47]) with established validity evidence.

***R1.5.*** *The study provides interesting insights into the factors linked to psychological stress, but the methodological gaps, especially around cross-validation and model evaluation, weaken the findings. Making these improvements in future revisions would boost the reliability and impact of the study*

***R1.5 Response.*** Thank you for these suggestions. They have greatly improved the strength of this manuscript.

[The following pages include responses to the comments from the journal requirements provided by the academic editor related to standards and requirements for publication in *PLOS ONE.*]

**Journal Requirements**

***JR.1*** *Please ensure that your manuscript meets PLOS ONE's style requirements, including those for file naming. The PLOS ONE style templates can be found at*

[*https://journals.plos.org/plosone/s/file?id=wjVg/PLOSOne_formatting_sample_main_body.pdf*](https://journals.plos.org/plosone/s/file?id=wjVg/PLOSOne_formatting_sample_main_body.pdf) *and*
[*https://journals.plos.org/plosone/s/file?id=ba62/PLOSOne_formatting_sample_title_authors_affiliations.pdf*](https://journals.plos.org/plosone/s/file?id=ba62/PLOSOne_formatting_sample_title_authors_affiliations.pdf)*.*

***JR1.1 Response.*** We apologize for this oversight, the manuscript and titlepage has been revised, as has the naming convention for files supplemental files.

*2. We noted in your submission details that a portion of your manuscript may have been presented or published elsewhere. [This manuscript is a revision of a thesis which was posted in UBC's public repository.] Please clarify whether this [conference proceeding or publication] was peer-reviewed and formally published. If this work was previously peer-reviewed and published, in the cover letter please provide the reason that this work does not constitute dual publication and should be included in the current manuscript.*

***JR1.2 Response.*** The original version was my MSc thesis. In accordance with University of British Columbia policy, all theses must be uploaded into their repository, cIRcle. This thesis never went through peer-review nor has it been formally published, and therefore does not constitute a dual publication.

*3. We note that you have indicated that there are restrictions to data sharing for this study. For studies involving human research participant data or other sensitive data, we encourage authors to share de-identified or anonymized data. However, when data cannot be publicly shared for ethical reasons, we allow authors to make their data sets available upon request. For information on unacceptable data access restrictions, please see* [*http://journals.plos.org/plosone/s/data-availability#loc-unacceptable-data-access-restrictions*](http://journals.plos.org/plosone/s/data-availability#loc-unacceptable-data-access-restrictions)*.*

*Before we proceed with your manuscript, please address the following prompts:*

*a) If there are ethical or legal restrictions on sharing a de-identified data set, please explain them in detail (e.g., data contain potentially identifying or sensitive patient information, data are owned by a third-party organization, etc.) and who has imposed them (e.g., a Research Ethics Committee or Institutional Review Board, etc.). Please also provide contact information for a data access committee, ethics committee, or other institutional body to which data requests may be sent.*

*b) If there are no restrictions, please upload the minimal anonymized data set necessary to replicate your study findings to a stable, public repository and provide us with the relevant URLs, DOIs, or accession numbers. Please see* [*http://www.bmj.com/content/340/bmj.c181.long*](http://www.bmj.com/content/340/bmj.c181.long) *for guidelines on how to de-identify and prepare clinical data for publication. For a list of recommended repositories, please see* [*https://journals.plos.org/plosone/s/recommended-repositories*](https://journals.plos.org/plosone/s/recommended-repositories)*. You also have the option of uploading the data as Supporting Information files, but we would recommend depositing data directly to a data repository if possible.*

*Please update your Data Availability statement in the submission form accordingly.*

***JR1.3 Response.*** We are not the legal owners of the data, which comes from Statistics Canada. However, after consulting with the data managers, we have been informed that the license used to acquire the data allows for data sharing. We have uploaded the original, raw data used for this project, and R syntax to Borealis and included the DOIs & URLs. Our Data Availability has been updated. Additionally, we added following a data availability section (p. 11, lines: 205-206):

The data included in the present analysis can be accessed through Borealis [34 ].

This citation links to the publicly available dataset and syntax used in the present analysis:

Hives BA. Multidimensional Correlates of Psychological Stress. UBC Borealis; 2025. doi:https://doi.org/10.5683/SP3/AKDVSL

*4. Thank you for uploading your study's underlying data set. Unfortunately, the repository you have noted in your Data Availability statement does not qualify as an acceptable data repository according to PLOS's standards.*

*At this time, please upload the minimal data set necessary to replicate your study's findings to a stable, public repository (such as figshare or Dryad) and provide us with the relevant URLs, DOIs, or accession numbers that may be used to access these data. For a list of recommended repositories and additional information on PLOS standards for data deposition, please see* [*https://journals.plos.org/plosone/s/recommended-repositories*](https://track.editorialmanager.com/CL0/https:%2F%2Fjournals.plos.org%2Fplosone%2Fs%2Frecommended-repositories/1/010f01944360ae3d-a21de4e2-04be-4c86-8c1c-c2f0e53602df-000000/0gWQuiSXmJEnCCrQ9N6Nzumlltf-PiXODW0m55gPAfk=192)*.*

***JR1.4 Response.*** See JR1.3 response. The files (i.e., data and R syntax) have now been uploaded to Borealis and the relevant DOI has been added to the manuscript.

*5. We notice that your supplementary figures are uploaded with the file type 'Other'. Please amend the file type to 'Supporting Information'. Please ensure that each Supporting Information file has a legend listed in the manuscript after the references list.*

***JR1.5 Response.*** Thank you for catching this error. We have corrected the file labelling and ensured that all files are included in the manuscript.
